# Educational strategies to train health care professionals across the education continuum on the process of frailty prevention and frailty management: a systematic review

**DOI:** 10.1007/s40520-018-0918-9

**Published:** 2018-02-23

**Authors:** Thomas Windhaber, Maria Lamprini Koula, Evangelia Ntzani, Alexandra Velivasi, Evangelos Rizos, Michail Theofilos Doumas, Evangelos Elias Pappas, Graziano Onder, Davide Liborio Vetrano, Angel Roudriguez Laso, Leocadio Roudriguez Manjas, Maddalena Illario, Regina Elisabeth Roller-Wirnsberger, On behalf of ADVANTAGE JA WP8 group

**Affiliations:** 1 Department of Internal Medicine, Medical University of Graz, Graz, Austria; 2 Society of Psychosocial Research and Intervention, Ioannina, Greece; 3 Department of Hygiene and Epidemiology, University of Ioannina School of Medicine, Ioannina, Greece; 4 Center for Evidence Synthesis in Health, Department of Health Services, Policy and Practice, School of Public Health, Brown University, Rhode Island, USA; 5 Department of Internal Medicine, University Hospital of Ioannina, Ioannina, Greece; 6 School of Medicine, University of Cyprus, Nicosia, Cyprus; 7 Department of Geriatrics, Università Cattolica del Sacro Cuore, Rome, Italy; 8 Aging Research Center, Department of Neurobiology, Care Sciences and Society, Karolinska Institutet-Stockholm University, Stockholm, Sweden; 9 Biomedical Research Foundation of the University Hospital of Getafe, Madrid, Spain; 10 Head of the Department Hospital Universitario de Getafe, Geriatrics, Madrid, Spain; 11 Campania Region Health Directorate, Division on Health Innovation, Federico II University and Hospital, Naples, Italy

**Keywords:** Frailty, Education, Training, Health care professionals, Health workers

## Abstract

**Background:**

In addition to the normal process of ageing, frailty, defined as a geriatric syndrome, is becoming more prevalent. Around 10% of people over 65 years and 25–50% of those aged over 85 years are frail. Frail elderly are more vulnerable to external stressors and have an increased risk of adverse health outcomes. To tackle these challenges, European Union (EU) member states need to develop a health work force capable of the right skills mix. A goal-centred education and training of professionals is crucial for effective and efficient health care delivery for Europe’s greying population.

**Aims:**

The aim of this study was to systematically collect, review and critically appraise studies carried out to investigate the efficacy and effectiveness of comprehensive educational programmes for health professionals related to frailty prevention and/or frailty management.

**Methods:**

A systematic review was carried out searching the databases PubMed, CINAHL, Cochrane CENTRAL, Medline, Up to date and Embase. Additionally, a manual search of the reference lists and searches via Google Scholar and greylit.org was done.

**Results:**

No relevant publications addressing the evidence and sustainability of educational/training programmes for frailty prevention and/or frailty management were identified.

**Discussion:**

The result of an empty review is surprising because several educational programmes in different countries are currently run.

**Conclusions:**

A significant knowledge gap exists in the scientific literature regarding education and training of health care workers regarding prevention and management of frailty. Further research is needed to identify effective educational strategies for health professionals to prevent and manage frailty.

## Introduction

The well-known demographic shift towards an increasing number of older people is well documented from the most developed countries to the lowest income regions. This presents challenges for societies [1, 2]. Functional decline and aged-related conditions are a major burden for older people, their families and health care systems [3]. In this context of understanding, frailty has become more common in addition to the normal ageing process. Around 10% of people over 65 years and 25–50% of those aged over 85 years are frail in accordance to the criteria established by Fried and colleges [4]. Frailty can be considered as a progressive age-related decline in physiological functions that results in higher vulnerability to external stressors [4, 5].

Frail older people are at increased risk of adverse health and social outcomes. Seemingly minor stressors may lead to serious health problems. Therefore, screening and monitoring for changes in individual resilience of older people is hallmark for early interventions to prevent a loss of functional and cognitive reserve and to maintain self-capacity for this numerically increasing number of older citizens [6, 7].

These challenges force care planners across all European Union (EU) member states to redirect their health care workforce capacities. Awareness, knowledge and skills among a large variety of professionals involved into the social and medical care process of older European citizens is, therefore, clue to develop an efficient and effective integrated frailty prevention approach (FPA) within member states of the EU [8]. Physicians, nurses and other medical staff need to be trained on detecting symptoms of pre-frailty and frailty and applying evidence-based interventions for prevention and management [4].

So far, only few studies have investigated the effectiveness of education and training programmes for health care professionals addressing older people’s functionality in different care settings [9–12]. To the best of the authors’ knowledge, no studies published in scientific literature provide an overview of education and/or training interventions for health care professionals in the field of frailty prevention. Therefore, the aim of this study was to systematically collect, review and critically appraise studies carried out to investigate the efficacy and effectiveness of educational programmes for health professionals related to frailty prevention and/or frailty management.

## Methods

The present study is characterized as a systematic review and the methods follow the guidelines from PRISMA [13].

### Data resources and search strategy

We searched PubMed, CINAHL, Cochrane CENTRAL, Medline, Up to date and Embase from February to May 2017, using English or another European language limit. Keywords were combined using Boolean operators and truncations. Several search algorithms were pilot tested starting with highly sensitive terms [14]. Given the observed difficulty to identify eligible studies, we chose to apply more lenient criteria for title/abstract screening opting for a large body of full texts for eligibility screening and thus enhanced sensitivity, we thus used general terms such as “worker” and “education” and the “explode” option so as to ensure that all MeSH terms would be included. The following search algorithm was finally adopted and implemented:

(frailty OR frail*) AND (education OR curriculum OR learning OR competence OR training) AND (“health worker” OR health-allied OR workforce OR professional OR physician OR worker).

Further, a manual search of reference lists of relevant papers and reviews was performed to identify additional articles. Besides, a web search was done on Google Scholar to a page depth of 12, using the keywords frailty, education and training. To identify grey literature, a search was conducted on greylit.org, using the same three keywords.

### Inclusion criteria

Studies had to meet the following criteria based on previously defined PICOT (Fig. 3 in “Appendix”) to be included into the search results:

- Quantitative research design.
- Explicitly addressing the education on frailty prevention and/or frailty management.
- Including professionals, health and social workforce working with older frail people.
- Providing educational/training models of health- and social care-related workforce/professionals (policy makers, physicians, nurses, nurse aids, social workers, dieticians, physiotherapists, occupational therapists and others) involved in the integrated care approach to prevent and/or manage frailty.
- Papers providing information concerning curriculum and competency development.
- A clear definition of frailty is stated.

Citations for eligibility were screened by two reviewers independently. Conflicts and disagreements were resolved by discussion with a third reviewer.

### Quality assessment

**Fig. 1:**
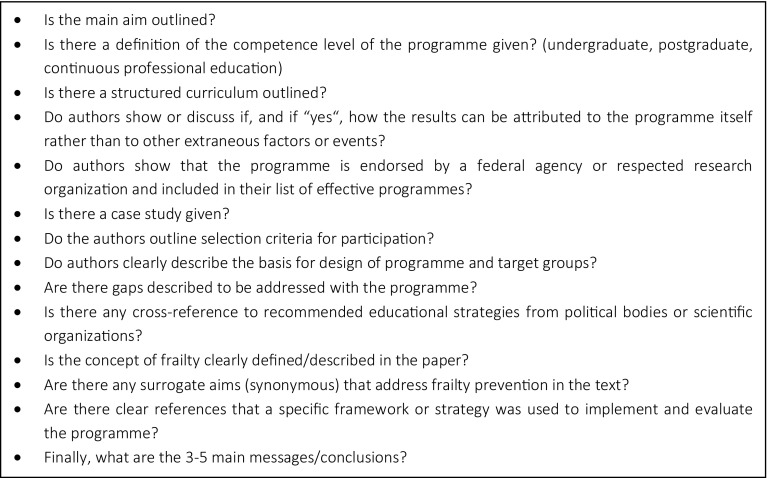
Quality indicators to further evaluate publications

To critically appraise the included studies, a template was developed by the authors. This template aims to gain information about quality indicators for education and training programmes recommended by the University of Wisconsin [15]. Furthermore, questions explicitly addressing the concept of frailty were included into the template. Quality indicators to further evaluate publications are listed in Fig. 1. Finally, a summary table of the quality is stated at the end of the questionnaire (Table 1).

**Table 1:** Summary table of the quality

	Yes	Partially	Don’t know	No
Case study				
The setting/context is clearly described				
The research question is well defined				
The methods are well described				
Results /data are systematically and stringently presented				
Interpretation is clearly based on the data				
There is a discussion of credibility and dependability of interpretation				
The results are presented in the context of previous research on the topic				

## Results

**Fig. 2:**
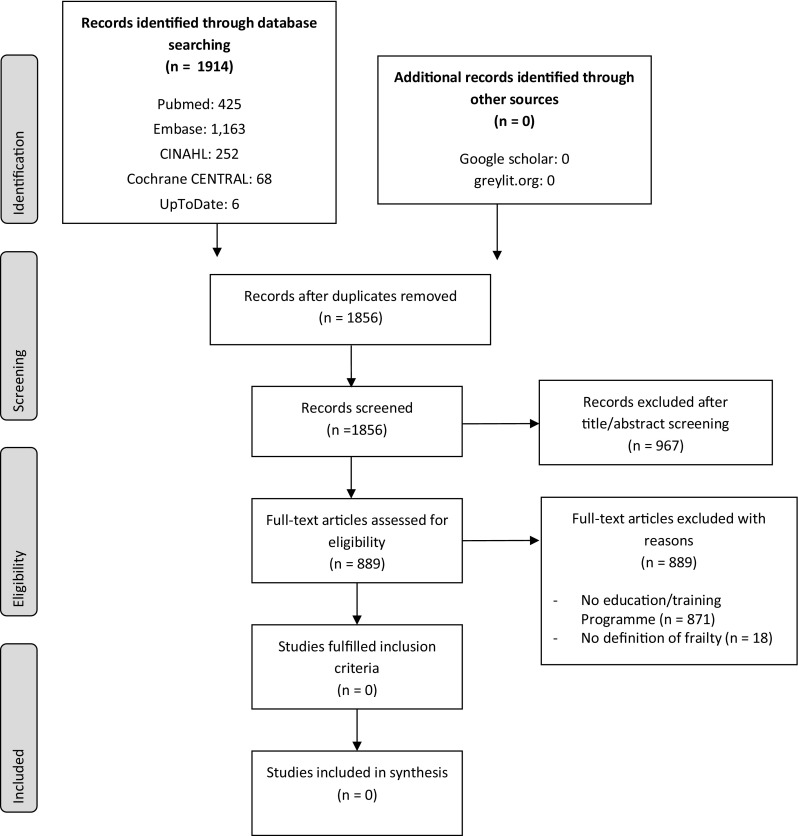
Flowchart of study selection, based on Moher et al. [31]

**Fig. 3:**
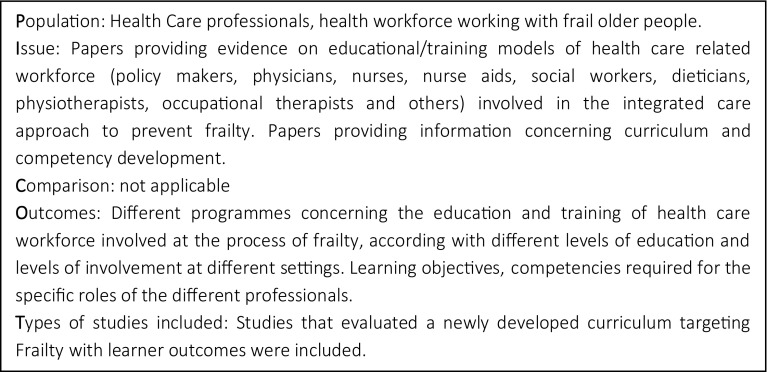
PICOT

Our comprehensive database search yielded 1,914 citations. No pertinent articles were retrieved from Google Scholar and greylit.org searches. Based on the algorithm outlined, 889 full-text articles were screened. Despite evaluation in depth (see Methods section), no publications addressing evidence and sustainability of educational/training programmes for frailty prevention were identified (see Fig. 2 PRISMA flowchart).

Studies were excluded for many reasons. The majority of articles did not address the search key criteria as they did not refer to educational programmes. Only three papers described educational programmes in the field of ageing and vulnerability, but frailty as a term was not explicitly defined or mentioned [16–18].

## Discussion

Evidence for education and training of health professionals in the field of frailty prevention and frailty management is scarce. In the comprehensive literature search presented in this study, no proof of efficacy or effectiveness of educational programmes for health care professionals to prevent frailty and functional decline of older people and published in international scientific literature could be detected. Complementary efforts to identify evidence through Google Scholar and grey literature resources were also not fruitful.

This result of an empty review is surprising in different ways:

Several educational programmes in different countries, also under the umbrella of national strategies, are currently run. All of those programmes aim at fostering self-care of citizens and their relatives and try to support prevention in the European member states. It is to be expected that the expertise gathered by professionals through attendance of those programmes has an effect in health care in the regions, provinces and on national level. However, none of the programmes found via search of grey literature has been evaluated in terms of sustainability on health care systems. Kirkpatrick’s evaluation would be one option to align evaluation of programmes in this context [19]. Using standardized evaluation offers the opportunity to tailor educational events for trainees needs and to adapt programmes to drive change management in health and social care across the systems [20]. As stated in the introduction of this paper, the demographic shift towards an ageing population fosters many health care systems to focus not only on active and healthy ageing and prevention of frailty and multimorbidity, but also older people care [6, 21]. In this context, frailty, pre-frailty and frailty prevention are key components of future needs in health care. Some European countries like Ireland have already realized this need and have set up national educational strategies to prepare health care workforce for this increasing demand in older care skills [22].

This observation is also in context with a publication recently launched by an expert group of geriatricians [23]. In this paper, geriatricians from across Europe outline the role and competences needed from medical doctors in older care in different care settings. Prevention on all public health levels is a key component needed from future medical doctors to tackle the demands raised by prolonged life expectancy of European citizens. Furthermore, the European Commission has funded three projects on frailty and frailty prevention also including work packages to collect information on educational opportunities for different professionals involved into older care in the member states involved into the projects [24–26]. Interestingly, none of these initiatives has so far launched or published evidence-based programme evaluation results.

The European Commission has recently launched a members states’ joint action (“Advantage”) aiming to build a common understanding on frailty on which to base a common approach to manage older people who are frail or at risk of being frail. This initiative is in line with the fact that preventing and managing frailty is a serious challenge for the public health sector [4, 27]. The development of health systems requires a multifaceted approach targeting resource deficits and the training and continuing education of sufficient personnel to meet elderly population needs [28]. Training health-related professionals on new clinical entities poses additional challenges as it requires not only reallocation of funds but also a change in mentality. In general, education and training of health professionals is key to ensure high-quality care of patients across all care settings [29].

The current publication is one of the first releases in the field of education and training arising from the Joint Action Advantage. The consortium of Advantage will release expert recommendations on skills and competencies needed for health care workforce to tackle the needs of health care systems in ageing societies [30]. The current review demonstrates the need for such approach due to lack of knowledge and evidence in medical education also for the frailty prevention approach.

### Strengths and limitations

Strength of the current study was the clear, broad and comprehensive search strategy to identify relevant articles. One limitation is the strict inclusion criteria. We chose to include only studies assessing educational programmes rather than single educational interventions which were beyond the scope of the present review. Broader criteria may have led to some publications addressing educational activities on frailty; nevertheless, such activities would not have the impact expected from programmes nor could be assessed using the same methodological framework.

## Conclusions

The aim of this study was to systematically collect, review and appraise studies carried out to investigate the efficacy and effectiveness of educational programmes for health professionals directly related to frailty prevention and/or frailty management. No relevant publications concerning education/training of health professionals in frailty prevention and/or frailty management were identified within this systematic review. Further research needs to evaluate programmes designed to train health professionals in the frailty continuum to gain knowledge about efficacy and effectiveness and give evidence-based recommendations on curricula development, structure and design. Education and training is, therefore, representing a big gap when building the approach for frailty prevention and management and a field of demand for further investigation.

## Appendix

See Fig. 3.
